# A dual-function epidermal growth factor receptor pathway substrate 8 (Eps8)-derived peptide exhibits a potent cytotoxic T lymphocyte-activating effect and a specific inhibitory activity

**DOI:** 10.1038/s41419-018-0420-5

**Published:** 2018-03-07

**Authors:** Xiaoling Xie, Weijun Zhou, Yuxing Hu, Yiran Chen, Honghao Zhang, Yuhua Li

**Affiliations:** Department of Hematology, Zhujiang Hospital, Southern Medical University, No. 253 GongyeDadaoZhong, Guangzhou, Guangdong 510282 China

## Abstract

The identification and characterization of tumor-associated antigens (TAAs) that generate specific cytotoxic T lymphocytes (CTLs) are vital to the development of cancer immunotherapy. The epidermal growth factor receptor (EGFR) pathway substrate 8 gene (Eps8) is involved in regulating cancer progression and might be an ideal antigen. In this study, we searched for novel human leukocyte antigen (HLA)-A*2402-restricted epitopes derived from the Eps8 protein via the HLA-binding prediction algorithm. Among four candidates, peptides 327 (EFLDCFQKF), 534 (KYAKSKYDF) and 755 (LFSLNKDEL) induced peptide-specific CTLs to secrete higher levels of interferon-gamma (IFN-γ) and showed enhanced cytotoxic activity against malignant cancer cells. Our results demonstrated that peptide-specific CTLs showed effective antitumor responses, including upregulation of interleukin-2 (IL-2), tumor necrosis factor-alpha (TNF-α), granzyme B and perforin. Treatment with peptide-sensitized peripheral blood mononuclear cells (PBMCs) significantly reduced the tumor growth in vivo compared with the non-peptide-sensitized PBMC treatment. Importantly, our results indicated that peptide 327 may interfere with EGFR signaling by mechanistically disrupting Eps8/EGFR complex formation. We extended this observation that peptide 327 also suppressed the viability of cancer cells, blocked EGFR signal pathway and reduced the expression of downstream targets. Notably, conjugation of peptide 327 to the TAT sequence (TAT-327) resulted in potent antitumor activity and selective insertion into cancer cell membranes, where it adopted a punctate distribution. Furthermore, peptide 327 and TAT-327 displayed anticancer properties in xenograft models. Our results indicated that 327, 534 and 755 were novel HLA-A*2402-restricted epitopes from Eps8. By inhibiting the Eps8/EGFR interaction, peptide 327 and TAT-327 may serve as novel peptide inhibitors, which could provide an innovative approach for treating various cancers.

## Introduction

Cancer is highlighted by the accumulation of a number of genetic variations and the loss of normal cellular regulatory processes and is a main cause of death worldwide^1^. Although modern therapies have prolonged the survival time of patients compared to that with traditional remedies, the vast majority of cancers remain incurable. Thus, the development of novel therapeutic modalities to improve survival rates is in great need^2^. Immunotherapy is a promising cancer treatment that has emerged with remarkable clinical efficacy, with the evidence that host immune responses can influence patient survival^3,4^.

Tumor-associated antigens (TAAs) are frequently present on various tumor cells but are absent or present at very low levels on normal cells and can be recognized by cytotoxic T lymphocytes (CTLs)^5,6^, leading to cytotoxic cellular responses^7^. Over the last few years, the identification of TAAs that are recognized by T cells has rapidly developed, in part due to advances in cancer immunology. While peptide vaccines that elicit a tumor-reactive immune response to TAAs have been under intensive investigation for decades, the number of antigens identified and the efficacy in clinical trials was previously limited^8^.

The epidermal growth factor receptor (EGFR) pathway substrate 8 (Eps8) is a TAA that is frequently overexpressed in breast, colon, and pancreatic cancers and other malignancies but rarely in normal tissues^9–12^. The *Eps8* gene was originally identified as a substrate for the EGFR kinase, which is known to promote tumor progression through an EGFR-dependent pathway. Furthermore, its aberrant expression often suggests an unfavorable prognosis for cancer patients^13–15^. Therefore, Eps8 has been considered an attractive target for specific cancer immunotherapy.

In the present study, we focused on Eps8 as a promising tumor antigen that drives induction of CTL responses against cancer cells. The use of peptide-based vaccines is a powerful and promising method to induce antigen-specific CTLs in cancer patients, and several clinical trials have been carried out^16–18^. Human leukocyte antigen (HLA)-A2 is the dominant type in Caucasians; therefore, HLA-A2-restricted peptide-based cancer immunotherapy has mainly been performed^19,20^. However, in Asia, HLA-A24 is more common, and clinical immunotherapeutic trials using specific HLA-A24-restricted peptides such as CEA, p53, PSMA, NY-ESO-1, and MAGE-A1 have been performed^21–24^. We have investigated three Eps8-derived peptides restricted to HLA-A*2402 epitopes using bioinformatics software and analyzed their potential as new immunotherapy epitopes. In this report, we showed that the 9-amino acid (aa) peptide 327, which partly mimics the EGFR binding region of Eps8, functions as a protein−protein interaction module that could disrupt the Eps8/EGFR complex and prevent the EGFR downstream pathway. To impart cell permeability to peptide 327, we linked peptide 327 to a cell-penetrating peptide, TAT, and designed a new cell-permeable derivative of peptide 327, herein named TAT-327^25^. We found that the addition of TAT could promote cellular uptake and TAT-327 was able to inhibit tumor growth in vitro and in vivo. Taken together, we report the screening and identification of HLA-A*2402-restricted epitopes and evaluated the potential of peptide 327 as an inhibitor of the Eps8/EGFR complex in vitro and in tumor-bearing mice. These findings support that the identified peptides can be utilized as novel strategies for a variety of cancers.

## Results

### Screening of HLA-A*2402-restricted Eps8-derived peptides

**Fig. 1:**
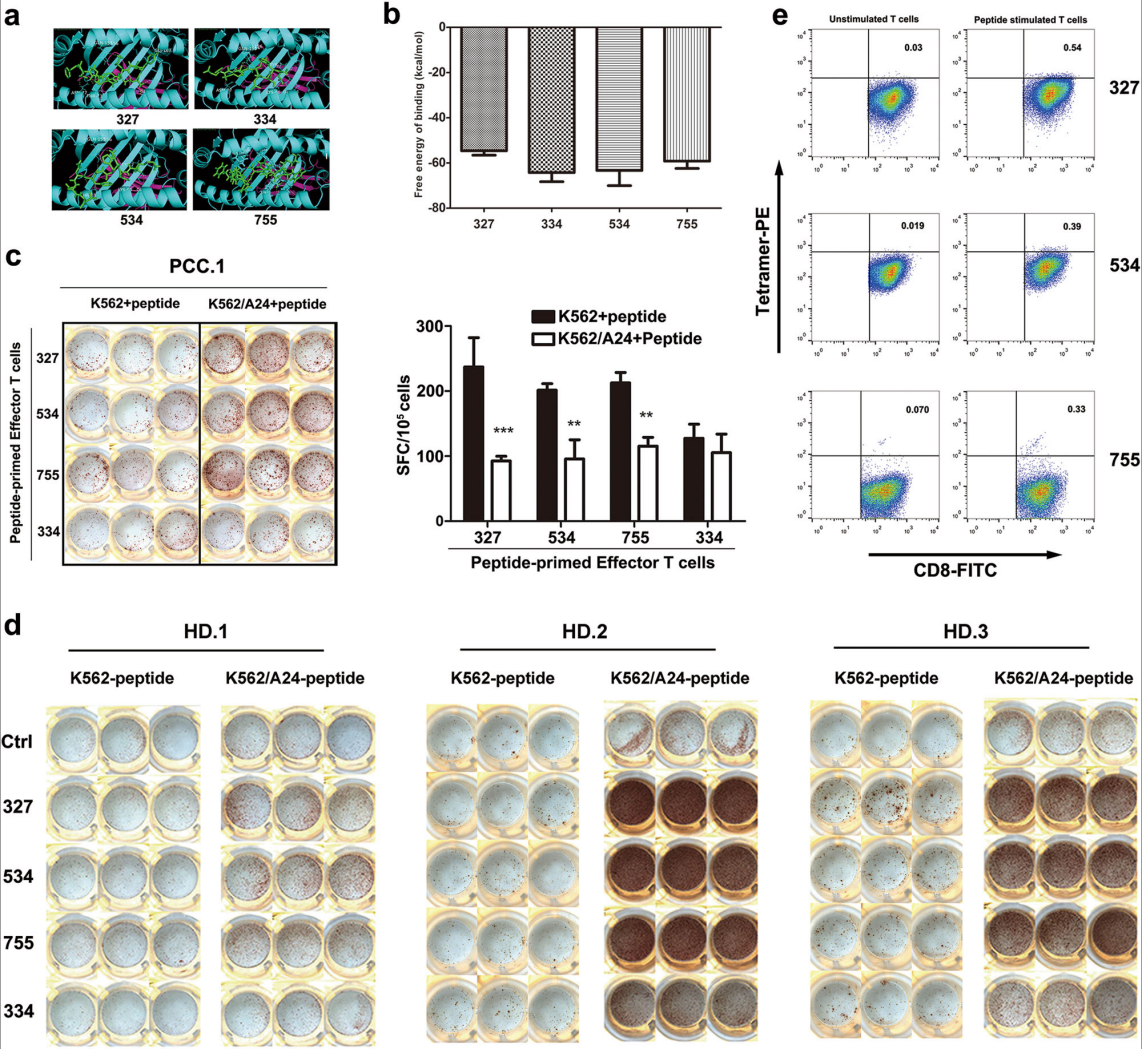
Identification of HLA-A24-restricted, Eps8-derived peptides. **a** Docked complex of HLA-A24 protein and a candidate peptide. PyMol (Molecular Graphics System) software was used to visualize this model. **b** Results for the free energy prediction. **c** Eps8 peptide-reactive CTLs were generated from HLA-A24+ prostate cancer patients. After three rounds of in vitro peptide stimulation (10 μM), peptide-sensitized PBMCs were examined by an ELISPOT assay using K562 or K562/A24 cells pulsed with corresponding peptide. The cells were cultured in triplicate. **d** Peptide-specific CTLs were generated from HLA-A*1101/2402+ HD.1, HLA-A*0207/2402+ HD.2, and HLA-A*1101/2402+ HD.3. The number of IFN-γ-producing CTLs was analyzed by ELISPOT assay. **e** Eps8-specific CD8+ T cells were measured using the PE-labeled HLA-A*2402/Eps8 tetramer along with APC-labeled anti-CD3 and FITC-labeled anti-CD8 mAbs

The aa sequence of the Eps8 protein was screened for the most likely HLA-A*2402 nonamer peptide epitopes using the SYFPEITHI and the BIMAS programs available online. We synthesized four nonamer peptides (327, EFLDCFQKF; 534, KYAKSKYDF; 334, KFKHGFNLL; and 755, LFSLNKDEL), corresponding to the Eps8 aa sequence that might bind to HLA-A*2402 as determined by epitope prediction algorithms (Table 1). Meanwhile, docking scores obtained from Autodock for candidate epitopes showed stable interaction of peptide-HLA-A24 complexes. The calculated free energy of binding (ΔG binding) displayed a strong affinity of the candidate peptides for the HLA-A24 protein (ΔG = −65 ~ −55 kJ/mol). As a representation, peptides docked with HLA-A24 allele using AutoDock and PyMol was displayed (Fig. 1a, b).

**Table 1:** Characterization of HLA-A*2402-binding affinity of Eps8-derived peptides

Peptide name	Amino acid	Position	BIMAS	SYFPEITHI
327	EFLDCFQKF	327–335	28.512	22
534	KYAKSKYDF	534–542	200.000	22
334	KFKHGFNLL	334–342	57.600	19
755	LFSLNKDEL	755–763	22.000	16

Nonamer peptides derived from Eps8 were chosen based on predicted binding affinity with the method of BIMAS and SYFPEITHI

### Natural T-cell responses against Eps8

The immune response of T cells to K562 and K562/A24 cells treated with Eps8-derived peptides 327, 534, 334 or 755 (10 μM) was assessed by interferon-gamma (IFN-γ) enzyme-linked immunospot (ELISPOT) assays after we isolated peripheral blood mononuclear cells (PBMCs) from five healthy individuals as well as four patients with cancer (Supplementary Tables 1 and 2). The results in Fig. 1c exemplify that the CTLs induced by all four of the peptides could produce IFN-γ in the patient with prostate cell carcinoma (PCC.1), while K562/A24 cells treated with peptides 327, 534 and 755 induced significantly higher IFN-γ production than the 334 peptide group (*P* < 0.01). Frequent and strong responses against 327, 534 and 755 were also detected in three healthy donors (Fig. 1d). Therefore, peptides 327, 534 and 755 were selected for further analysis. Consequently, the HLA-A*2402/Eps8 tetramer was prepared and used to confirm the frequency of Eps8-specific CD8-positive (+) T cells. PBMCs from HLA-A24+ donors were stained with the HLA-A*2402/327 (EFLDCFQKF), HLA-A*2402/534 (KYAKSKYDF) or HLA-A*2402/755 (LFSLNKDEL) tetramer. On average, 0.3% of the CD8+ T cells were Eps8-specific CD8+ T cells. In contrast, almost no Eps8-specific T cells were detected in the PBMCs without peptide stimulation (Fig. 1e).

**Fig. 2:**
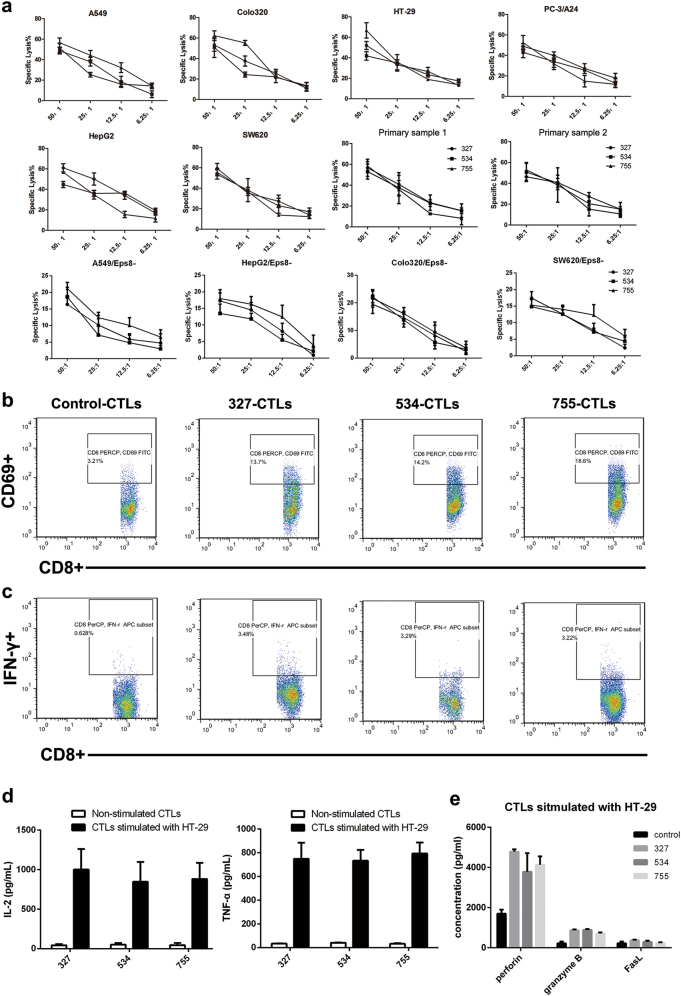
Peptide-specific CTLs exhibit CTL-activating effects on various cancer cells. **a** CTLs were subjected to A549, Colo320, HepG2, SW620, PC-3/A24, HT-29 cells and patient-derived tumor cells (HLA-A24+, Eps8+), as well as Eps8-silenced cells (HLA-A24+, Eps8-) at the indicated effector/target ratios (50:1, 25:1, 12.5:1, 6.25:1) by means of a standard LDH release assay. **b** The CD69 expression on the cell surface was evaluated after peptide stimulation by flow cytometry. **c** Representative flow cytometric analysis demonstrates increased IFN-γ by CTL in response to HLA-A24+ cancer cells (HT-29). CTLs showed a significantly higher level of IFN-γ secretion (*P < 0.05) in response to Eps8+ and HLA-A2+ cancer cells as compared to that in response to control CTLs cultured in media alone. **d**, **e** IL-2, TNF-α, granzyme B, perforin and FasL secretion levels by CTLs were measured by ELISAs in the cultured supernatants collected 24 h following stimulation with tumor cell lines

Next, the cytotoxic activities of the three peptides were examined. The cancer cell lines A549, Colo320, HepG2, HT-29 and SW620 were Eps8+ and HLA-A24+ (Supplementary Figure 1). The A24-negative PC-3 cell line was transfected with HLA-A24. We also performed cytotoxic assays using patient-derived tumor cells as target cells. As indicated in Fig. 2a, Eps8-reactive CTLs stimulated with 327, 534, or 755 peptides exhibited effective cytotoxicity to A549, Colo320, HepG2, SW620, PC-3/A24 and HT-29 cells that were Eps8+ and HLA-A24+ at all effector cell to target cell (E:T) ratios (6.25, 12.5, 25 and 50). Results also showed that peptide-primed CTLs cultured from PBMCs of HLA-A*2402-positive donor (PCC.1 and PCC.4) lysed primary cells derived from the same patients. We also included a control group with four Eps8-unexpressed cell lines to confirm the specificity of the killing activity. We found that the Eps8-CTLs produced less cytotoxicity to cells silencing Eps8. These results suggest that the epitope peptides 327, 534 and 755 are potential immunogenic Eps8-derived epitopes for HLA-A*2402 subjects.

### Peptide-specific CTLs effectively induce and expand CD3+ CD8+ CTLs from T cells of HLA-A24+ donors, and the CTLs display cytokine production in response to cancer cell lines

The resulting CTLs were evaluated for the frequency of CD3+ CD8+ T cells by flow cytometry at 1 week after the first stimulation. The peptide-sensitized CTLs contained a higher proportion of CD3+ CD8+ T cells (>40%) than the unstimulated T cells after the first stimulation (Supplementary Figure 2). Next, we analyzed the portion of activated cells (% of CD69+ cells of the CD3+ CD8+ T cells), and the 327-CTLs, 534-CTLs, and 755-CTLs demonstrated an increased proportion of CD69+ CD3+ CD8+ T cells (Fig. 2b). We further analyzed the 327-CTLs, 534-CTLs, and 755-CTLs for their antitumor activities, including the level of IFN-γ production in response to cancer cell lines. Representative flow cytometric analyses demonstrated that the 327-CTLs, 534-CTLs, and 755-CTLs had specific intracellular IFN-γ production against the HLA-A24+ cell lines (Fig. 2c).

To test the immunogenicity of these peptides, the Eps8-derived peptide-stimulated CTLs were evaluated for their ability to release interleukin-2 (IL-2) and tumor necrosis factor-alpha (TNF-α) upon stimulation in HT-29 cells. Enzyme-linked immunosorbent assay (ELISA) was used to determine the cytokine release and further confirmed that the peptide-sensitized CTLs induced specific T-cell responses. These data showed that the secreted levels of IL-2 and TNF-α were positively correlated with cytotoxicity against HT-29 cells (Fig. 2d). The concentration of three cytotoxic effector molecules including perforin, granzyme B, and FasL in co-culture supernatants was also measured by ELISA. Results showed that the peptide-stimulated group had higher levels of perforin and granzyme B, while non-peptide stimulated groups had positive, but lower levels. As seen in Fig. 2e and Table 2, the peptide-stimulated and non-peptide-stimulated groups showed no differences for FasL expression. It was initially considered that tumor cells are killed by granzyme B and perforin secretion.

**Table 2:** Level of different cytokine in various peptide group ($\overline x$ ± s, pg/mL)

Group	Control	327	534	755
granzyme B	221.20 ± 89.96	868.37 ± 45.69	888.55 ± 44.00	709.21 ± 54.771
perforin	1698.25 ± 201.36	4781.65 ± 119.54	3784.78 ± 928.67	4134.23 ± 419.44
FasL	224.40 ± 90.41	371.44 ± 31.01	294.45 ± 56.20	233.28 ± 38.68

### Efficacy of peptide-sensitized CTLs in an animal model

**Fig. 3:**
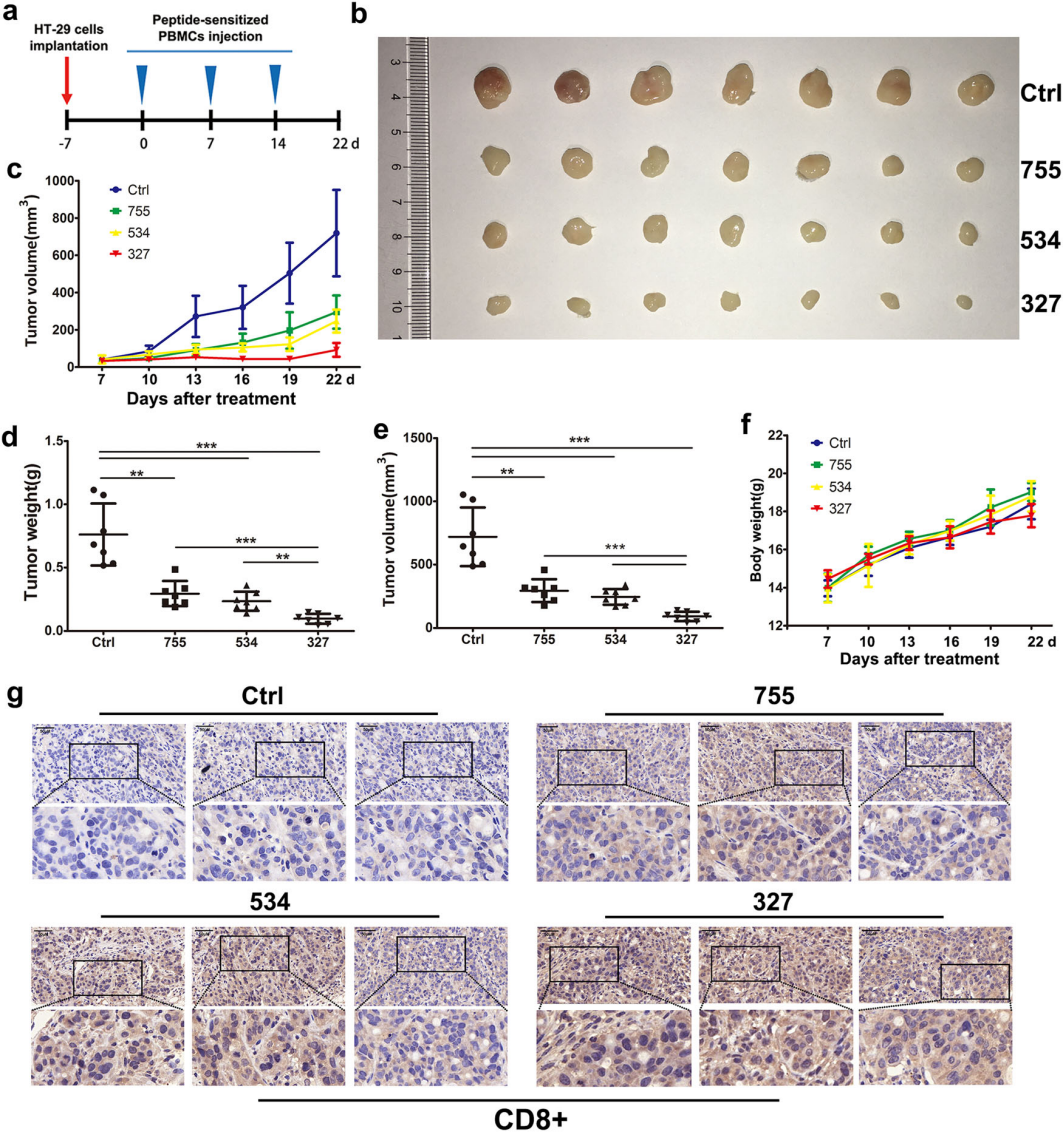
Therapeutic effects of transplanted 327-sensitized, 534-sensitized, and 755-sensitized CTLs in mice bearing HT-29 xenografts. **a** The schema HT-29 cell inoculation and injection of PBMCs. **b** Groups treated with peptide-sensitized CTLs showed a greater decrease in tumor volume than the control group treated with non-peptide-stimulated T cells. Points, mean (*n* = 7); bars, standard deviation (SD). **c** At the end of the experiment, tumors were removed, and images were taken with a digital camera. Tumor volume (**d**) and tumor weights (**e**) of the killed mice were evaluated 29 days after tumor implantation. **f** Effect of CTLs on mouse body weights. **g** Images from immunohistochemistry of CD8+ staining indicated that CD8+ CTLs successfully infiltrated into tumor tissues of the experimental groups

To investigate whether 327-sensitized, 534-sensitized, and 755-sensitized CTLs could show a potent therapeutic effect against human cancer in vivo, we constructed a mouse model by subcutaneous injection of HT-29 cells into BALB/c nude mice. Mice in the experimental groups were injected once a week for three consecutive weeks with 1×10^7^ CTLs that were isolated and sensitized with peptides 327, 534, or 755 (Fig. 3a). By day 29, mice in the experimental groups showed significant tumor suppression compared to those in the control group treated with non-peptide-sensitized CTLs (Fig. 3b, c, e). Compared with control mice, the peptide-sensitized groups showed a greater reduction in tumor weight (Fig. 3d). In addition, there was no obvious difference in body weight among the four groups (Fig. 3f). After the tumors were removed, we performed immunohistochemical analysis to determine the protein expression of CD8 in the tumor tissue. The results indicated that CD8+ CTLs infiltrated into the tumor tissues in the nude mice that were injected with 327-sensitized, 534-sensitized, or 755-sensitized CTLs as shown by the increased expression of CD8 in comparison to that in the tumor tissues from the vehicle group (Fig. 3g).

### A novel peptide inhibitor and its cell-permeable derivative

**Fig. 4:**
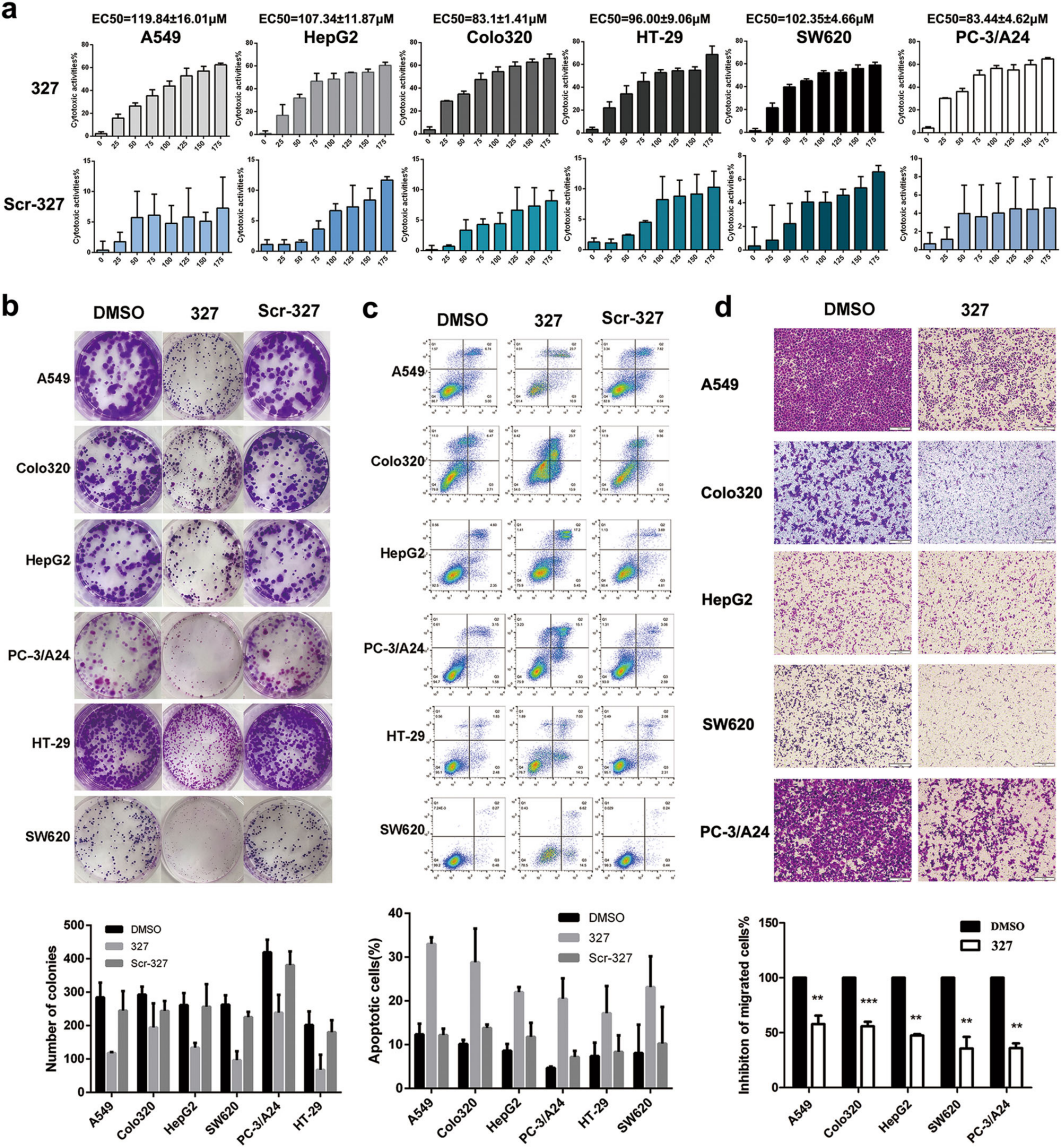
Peptide 327 inhibits tumor cell growth. **a** Cytotoxicity of 327 and Scr-327 toward various human cancer cells following treatment with peptide for 12 h, as measured by CCK-8 assays (*n* = 3). **b** Colony formation ability of cancer cells following treatment with peptide 327 and Scr-327 (100 μM). **c** Apoptosis of cancer cells treated with peptide 327 and Scr-327 (100 μM) for 12 h was assessed by using flow cytometry. Data obtained from the Annexin V-APC/PI double staining and flow cytometry assay shows apoptosis levels. **d** Migrated activity of cancer cells in the absence or presence of peptide 327 (100 μM) in the transwell assay, and the quantification of migrated cells through the membrane is shown as a proportion of their vector control (*n* = 3)

Previous studies have shown that Eps8 interacts with EGFR and triggers its downstream pathway. One peptide with the sequence positions 327 to 335 (EFLDCFQKF), termed 327, is located in the EGFR binding region for Eps8 (aa 289–362). We assumed that peptide 327 would interfere with the formation of the Eps8/EGFR complex by 327 mimicking the EGFR binding region on Eps8. We consider peptide 327 a novel peptide inhibitor that competes with the Eps8 protein and binds to EGFR. To explore the potential role of peptide 327 in modulating tumor growth, various cancer cell lines were treated with peptide 327 at the indicated concentrations (25, 50, 75, 100, 125, 150 and 175 μM) for 12 h, and the effects on cell proliferation were assessed by cell counting kit-8 (CCK-8) assays. We also included a scrambled peptide (Scr-327, LCEFDFKQF) as a control. As illustrated in Fig. 4a, peptide 327 significantly inhibited cell proliferation—in a dose-dependent manner. The half maximal effective concentration (EC_50_) values of peptide 327 are listed as follows for the different cell types: A549, 119.84 ± 16.01 μM; HepG2, 107.34 ± 11.87 μM; Colo320, 83.10 ± 1.41 μM; HT-29, 96.00 ± 9.06 μM; SW620, 102.35 ± 4.66 μM; and PC-3/A24, 83.44 ± 4.62 μM. The growth inhibition rates of Scr-327 were less than 10% in all tumor cells. We then examined the effect of peptide 327 on the colony-forming capability of the cancer cells and found that peptide 327 significantly reduced the colony numbers as shown by the representative colony growth on six-well plates compared to the growth with dimethyl sulphoxide (DMSO) and Scr-327 treatments (Fig. 4b). To quantify 327-induced apoptosis, various cancer cells were treated with peptide 327 for 12 h and stained with Annexin V and PI. As shown in Fig. 4c, after a 12 h exposure to 100 μM peptide 327, a significant increase in the apoptosis rate of the cells was observed. Collectively, these results indicated that peptide 327 effectively suppresses cell survival and promotes apoptosis. We then evaluated the effect of 327 on cell migration. The transwell assay demonstrated that 327-treated cells showed a reduced migration capability compared with that of the cells treated with DMSO (Fig. 4d).

### Peptide 327 blocks the Eps8/EGFR interaction and suppresses the EGFR downstream pathway in cancer cells

**Fig. 5:**
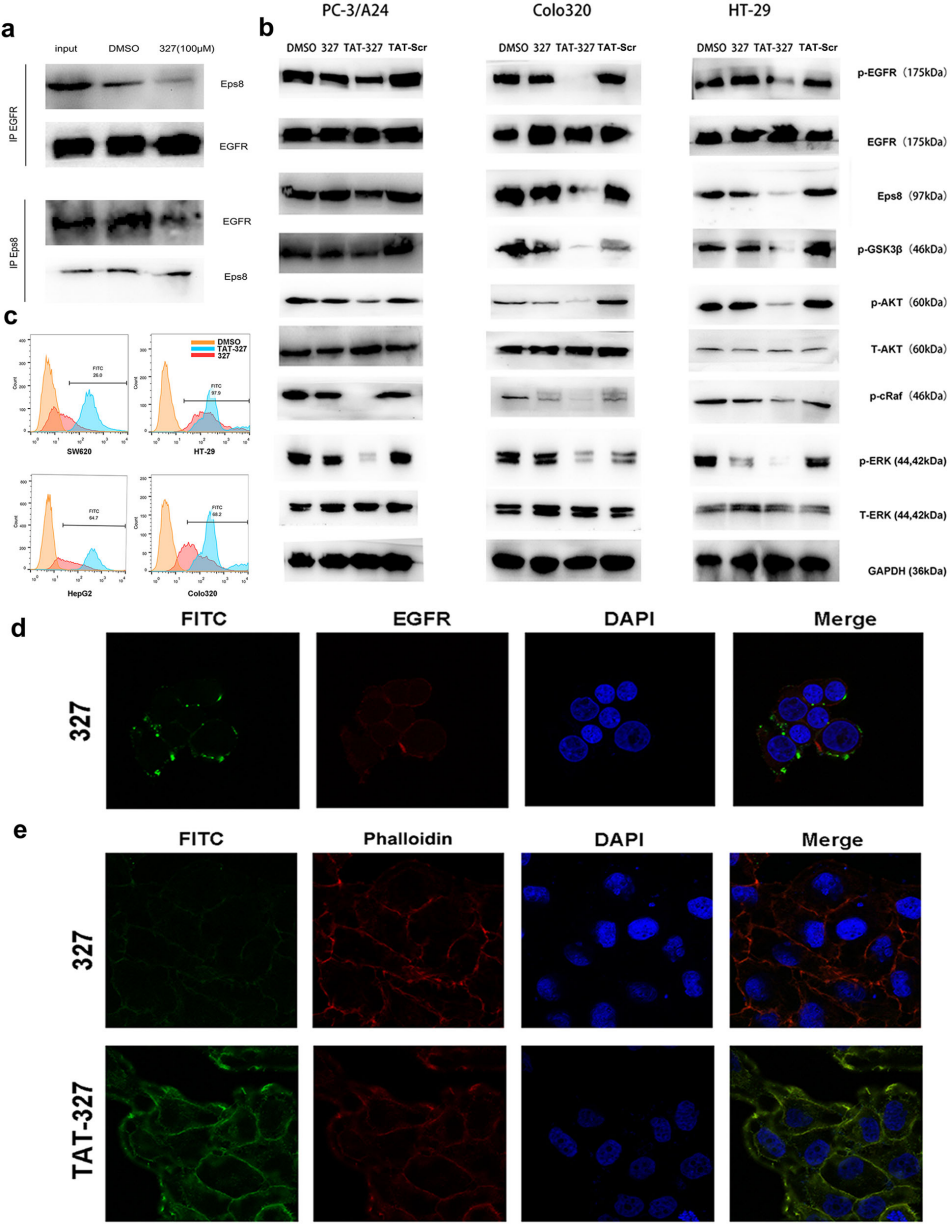
The peptide 327 blocks the Eps8/EGFR interaction. **a** The endogenous Eps8 interaction with EGFR is blocked by peptide 327. EGFR was immunoprecipitated from Colo320 protein extracts pretreated with 327 as indicated using an anti-EGFR antibody. Immunoprecipitants were analyzed by western blot using Eps8 or EGFR antibodies. The interaction was abrogated by pretreatment with 100 μM peptide 327. **b** Western blot assays for AKT, p-AKT, ERK, p-ERK, p-GSK3β, p-c-Raf, p-EGFR, EGFR and Eps8 in Colo320, HT-29 and PC-3/A24 cells following treatment with 327 and TAT-327 (50 μM) for 12 h. **c** Flow cytometric of the uptake of FITC-327 or FITC-TAT-327 in different cells. **d** Confocal images showed that peptide 327 (shown in green) was co-localized with EGFR (shown in red). **e** Confocal images of cancer cells treated with FITC-labeled 327 and TAT-327 (50 μM) for 2 h. TAT-327 was able to easily access the surface of the cells as compared to the peptide 327. Nuclei (DAPI, blue), peptide (FITC, green), cytoskeleton (Rhodamine-Phalloidin, red)

Since the EGFR binding region of Eps8 is predicted to target the juxtamembrane region of EGFR, peptide 327 (aa 327–335) derived from the EGFR binding region on Eps8 may disrupt the Eps8/EGFR interaction and lead to the dissociation of Eps8 from the Eps8/EGFR complex, subsequently downregulating the downstream pathway. EGFR is a transmembrane protein, and Eps8 is an intracellular protein. We assume that Eps8 interacts with EGFR in the juxtamembrane domain of EGFR (intracellular and close to the cell membrane). To test this hypothesis, Colo320 cells expressing high levels of endogenous Eps8 and EGFR were treated with 327 (100 µM) for 12 h. Co-immunoprecipitation was performed using an Eps8 or EGFR antibody. The results indicated that treatment with 327 resulted in Eps8/EGFR complex dissociation. As shown in Fig. 5a, only minimal amounts of Eps8 could be detected in the EGFR immunoprecipitants after treatment with peptide 327. Our results showed that 327 is a potent peptide inhibitor that was predicted to disrupt the Eps8/EGFR complex. Meanwhile, we performed EGFR and peptide 327 co-localization experiments to study whether 327 can actually bind to EGFR (Fig. 5d). Cells were treated with FITC-labeled peptide 327 (shown in green), the cells were then fixed, permeabilized, and incubated overnight with anti-EGFR antibody (pseudocolored in red). Nuclear DNA was labeled with DAPI (shown in blue). Image was taken and the results obtained from laser confocal scanning microscopy showed co-localization of peptide 327 and EGFR. Although peptide 327 was able to suppress the biological activity of cancer cells, we tried to improve its antitumor effect. Thus, we conjugated a cell-penetrating peptide, transactivator of transcription (TAT, RKKRRQRRR), to peptide 327 to impart cell permeability in the resulting peptide, TAT-327 (RKKRRQRRREFLDCFQKF).

To further examine the inhibitory effect of 327 and TAT-327 on EGFR downstream signaling, we detected the expression of the EGFR target proteins by western blotting. Cancer cells express high levels of Eps8 and EGFR among the cell lines that we have examined were treated with peptide 327 and TAT-327 (50 µM) for 12 h. We also included control groups treated with DMSO and TAT-Scr (scrambled peptide, RKKRRQRRRLCEFDFKQF). The western blotting results showed that the level of each protein expression was apparently decreased after treatment with TAT-327 compared with that after treatment with DMSO and TAT-Scr. As expected, cells treated with 327 did not show a significant change in the expression levels of these proteins in comparison with the cells treated with TAT-327 when using a relatively lower dose (50 µM) (Fig. 5b). We next investigated whether TAT-327 can penetrate the plasma membrane effectively. In the first set of experiments, we tested the affinity of FITC-labeled 327 and TAT-327 for different cancer cells. The fluorescence intensity of different cancer cells after 2 h treatment with the FITC-labeled TAT-327 was higher than that cells treated with the FITC-labeled 327 (Fig. 5c). These results were in accord with the confocal laser scanning microscopy results. Weak fluorescence was observed in the cells incubated with FITC-labeled 327. By contrast, the cells treated with FITC-labeled TAT-327 exhibited stronger fluorescence. As seen in Fig. 5e, TAT-327 localized in a punctate pattern at the plasma membrane of the cancerous Colo320 cells, with a diffuse intracellular distribution, indicating the occurrence of enhanced cell permeability. This difference in distribution suggests an accumulation for TAT-327 in the membrane and cytoplasm.

**Fig. 6:**
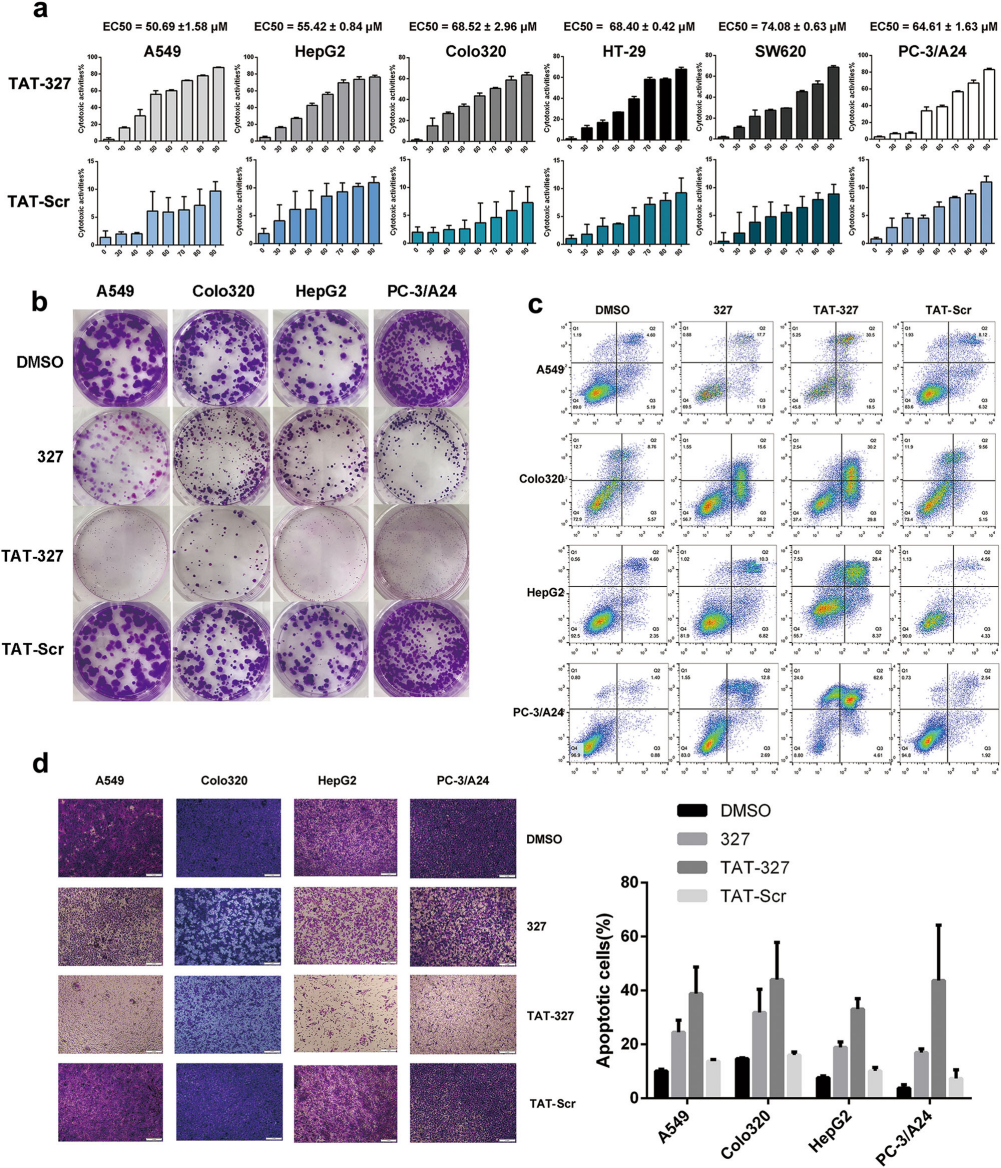
Biological activity of TAT-327 in vitro. **a** Cancer cell lines were treated with TAT-327 or TAT-Scr for 12 h at increasing concentrations (0, 30, 40, 50, 60, 70, 80 and 90 μM), followed by assessment of cell viability using CCK-8 assays (*n* = 3). **b** Treatment with TAT-327 significantly inhibited colony formation. **c** Cancer cells were incubated with DMSO, 327, TAT-327 or TAT-Scr for 12 h and then analyzed for apoptosis using Annexin V/PI staining assays. Quantification of Annexin V/PI staining is shown. **d** Cancer cells were treated with DMSO, 327, TAT-Scr or TAT-327 for 12 h in migration assays

The data presented so far have demonstrated that peptide 327 and TAT-327 effectively inhibited downstream targets of EGFR in cancer cells. Therefore, we next examined whether TAT-327 could also suppress tumor cell growth and migration. We treated cancer cells with increasing concentrations (0, 30, 40, 50, 60, 70, 80 and 90 μM) of TAT-327 for 12 h. The effect of the TAT-327 TAT-Scr peptide on the viability of cancer cells was determined using CCK-8 assays. TAT-327 induced a dose-dependent decrease in cell viability (*P* < 0.05; Fig. 6a). Colony formation assays were performed to test the inhibitory effect of peptide 327 and TAT-327 on cell proliferation, and similar results were obtained (Fig. 6b). Annexin V staining indicated that TAT-327 induced a higher rate of cellular apoptosis than peptide 327 in cancer cells after 12 h of incubation (Fig. 6c). The ability of TAT-327 to inhibit the migration of cancer cells was then examined by an in vitro scratch wound healing assay (Supplementary Figure 3). The untreated cells migrated within 24 h to close the wound, while TAT-327 treatment prevented this migration. Transwell assays showed that TAT-327 significantly reduced the number of migrated cells compared with DMSO, TAT-Scr or peptide 327, as revealed by crystal violet staining (Fig. 6d). These data suggest that TAT-327 may exhibit stronger antitumor activity than 327.

### Peptide inhibitor treatment inhibits tumor growth in xenograft mouse models

**Fig. 7:**
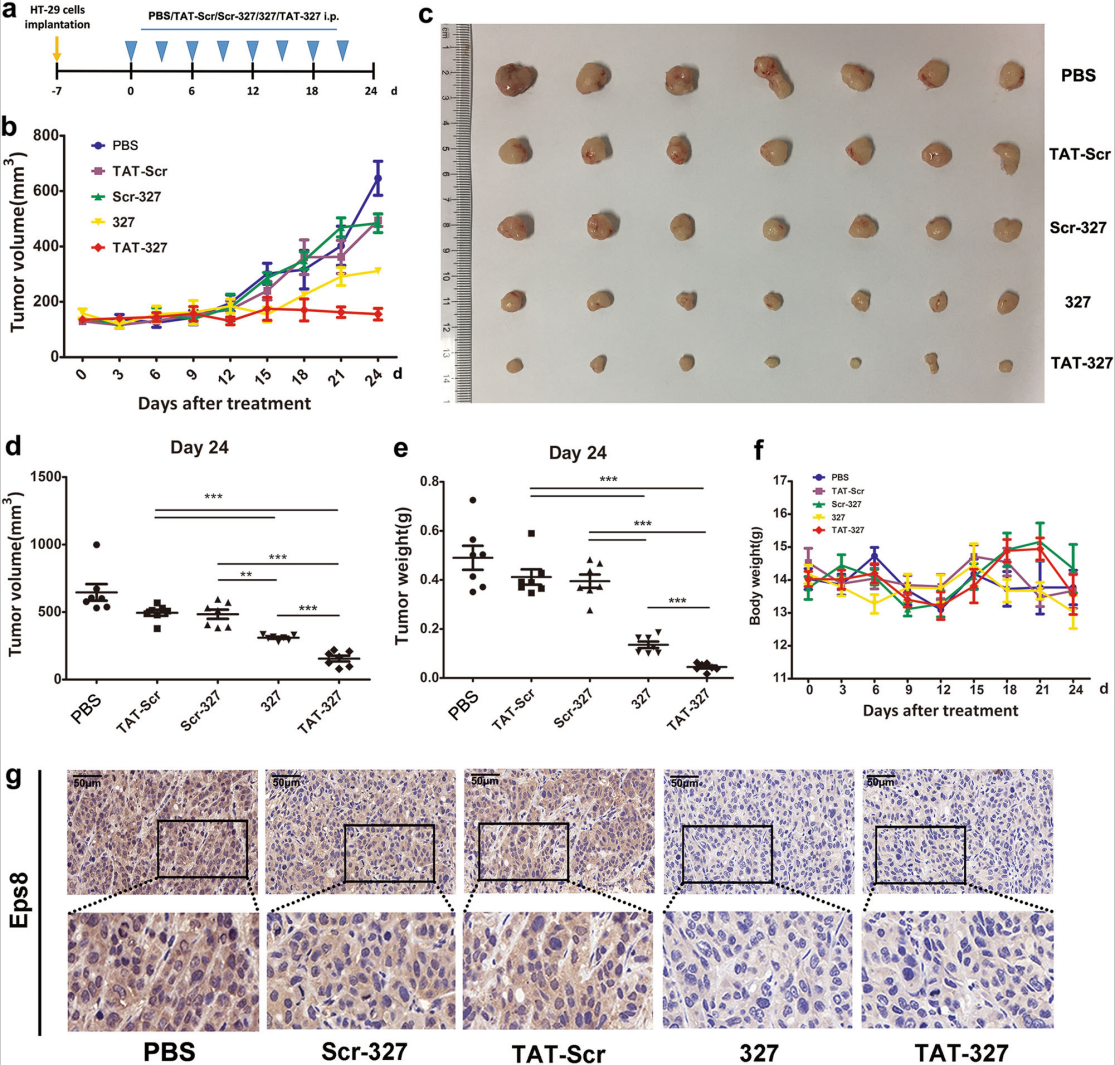
Antitumor activities of 327 and TAT-327 in an animal model. **a** Treatments were initiated 7 days after HT-29 cell transplantation when the tumors reached 50 mm^3^ and were administered by i.p. injection with 327, TAT-327, Scr-327, TAT-Scr or PBS. **b** Tumor volumes were measured using calipers. **c** A photograph of tumors excised from the mice after 24 days after treatment. **d**, **e** The mean tumor volume and weight are shown after the mice were killed. **f** The mean body weight (7 SDs of the mean) for each treatment group is shown. **g** Eps8 staining of a representative tumor section is shown for each treatment group

We next sought to explore if peptide 327 and TAT-327 exhibited anticancer activity in an orthotopic HT-29 xenograft mouse model. Compared to the PBS, Scr-327 and TAT-Scr treatments, the TAT-327 and 327 treatments significantly suppressed tumor growth, as shown by the photos of excised tumors at the end of the experiment (Fig. 7c). Both 327 and TAT-327 significantly inhibited tumor growth (*P* < 0.001) in HT-29 xenograft models compared to the other three treatments (Fig. 7b, d, e). We kept monitoring mouse body weights over a 24-day period to assess toxicity and found no statistically significant differences in mouse body weight among the three experimental groups, indicating that these peptide inhibitors have very low or no toxicity in vivo (Fig. 7f). As shown in Fig. 7g, tumors from the peptide inhibitor-treated mice showed decreased proliferation compared to those from the control mice, as assessed by Eps8 staining. These data demonstrate that peptide 327 and TAT-327 are potential peptide inhibitors that are able to reduce the growth of tumors.

## Discussion

Many HLA-A24-restricted CTL epitopes have been reported, and some have actually been utilized in clinical trials against solid cancers, as 20% of Caucasians, 12% of Africans and 60% of Japanese individuals (95% of whom have an A*2402 genotype) are positive for HLA-A24^26^. Compared with conventional therapy, immunotherapy has become an attractive therapeutic modality for cancer. Active cancer immunotherapy is based on the identification of immunogenic TAA epitopes to induce CTL responses. Notably, Eps8 plays an important role in driving tumor progression, making it an attractive target.

Few effective CTL epitopes derived from Eps8 have been identified to date, and those that have are restricted to HLA-A*0201 and HLA-A*1101^17,18,27^. Although HLA-A*2402 is one of the most common epitopes in different human races, HLA-A*2402-restricted Eps8 epitopes have not yet been reported. To identify Eps8 peptides specific to HLA-A*2402, we used the online software SYFPEITHI and BIMAS to predict peptides having a high affinity for HLA-A*2402. Based on our bioinformatics search, we selected and synthesized four Eps8-specific peptides. Among the peptides, 327 (EFLDCFQKF), 534 (KYAKSKYDF) and 755 (LFSLNKDEL) were highly immunogenic in human CD8+ T cells. Results showed that the peptide-sensitized CTLs had a higher CD69+ T-cell subset and produced IFN-γ in response to tumor cell recognition. Meanwhile, peptide-specific T cells could efficiently kill HT-29 cells, and produced IL-2, TNF-α, perforin, and granzyme B, with minimal production of FasL (Fig. 2 and Table 2). As expected, intratumoral injection of peptide-sensitized CTLs strongly inhibited tumor growth as detected by volume and weight. We also found that these mice tolerated the treatment well, as shown by no significant body weight loss after the treatment (Fig. 3), suggesting that these epitopes might be useful for immunotherapy in cancer patients.

Moreover, peptide 327, which spans the EGFR binding region of Eps8 and is responsible for the interaction with EGFR, exhibits antitumor properties. Therefore, we propose that the observed antitumor effect of peptide 327 can be explained by a competitive inhibition toward EGFR proteins for interaction with Eps8. Inhibitors that can block upstream or downstream elements in the EGFR signaling pathway have been extensively developed in cancer research over the past two decades because EGFR downstream signaling regulates tumor progression via proliferation, metastasis, angiogenesis, and drug resistance mechanisms. The juxtamembrane domain of EGFR^28,29^ is involved in the mechanism of receptor activation, and targeting this region could be a promising strategy. In our study, we investigated whether blocking the interaction of Eps8 with EGFR could have a biological effect in cancer cells. We hypothesized that this complex inhibitor can negatively affect the survival of cancer cells by suppressing the downstream signaling of EGFR. Our data support this hypothesis. We have demonstrated that the EGFR binding region of the Eps8 protein could be mimicked by a peptide encoding a portion of the EGFR binding aa sequence, which could potentially lead to decreased EGFR signaling at the protein level and consequently have inhibitory effects^30^. This supports our proposal for the therapeutic effects of Eps8/EGFR inhibitors on malignant cancers.

A relatively high dose of 327 is required to effectively inhibit the growth of cancer cells. Since peptides often require a vector to enhance cellular entry, we linked the HIV-1 TAT protein transduction domain (HIV-TAT 49–58) to peptide 327 to generate TAT-327^31–33^. Results showed that the addition of cell-penetrating TAT-327 enabled cellular uptake and consequently improved the efficiency of the peptide. The new cell-permeable inhibitor showed superior efficacy to 327 against cancer cells.

In summary, we screened and identified three immunogenic HLA-A24-restricted epitopes (327, 534, and 755), which can be used to generate CD8+ CTLs in vitro and in vivo. We also demonstrated that peptide 327, a specific Eps8/EGFR inhibitor, can compete with Eps8 in interacting with EGFR. Additionally, as expected for a cell-penetrating peptide, the resulting TAT-327 peptide accumulated in the cytoplasm more easily, thereby displaying anticancer activity.

## Materials and methods

### Study subjects

Five HLA-A*2402+ healthy volunteers and four HLA-A*2402+ prostate cancer patients were enrolled in this study after obtaining informed consents. High-resolution sequence-based typing of HLA was performed, and collection of blood samples was authorized by the ethical review board of Zhujiang Hospital (Guangzhou, China).

### Epitope prediction and peptide synthesis

Bioinformatic methods were used to predict and identify HLA-A*2402-restricted CTL epitopes from Eps8 as previously reported. In brief, a computational supermotif algorithm (SYFPEITHI, http://www.syfpeithi.de/) combined with a quantitative motif algorithm (BIMAS, https://www-bimas.cit.nih.gov/) were used for the prediction^34^. According to the two calculation scores, the top four candidate epitopes were verified and synthesized (Chinese Peptide, Hangzhou, China) and the purity was >95% for all synthetic peptides. Scrambled version of peptides 327 (Scr-327, LCEFDFKQF) was also prepared. All the peptides were dissolved in DMSO and stored at −80 °C until use^35^.

### Cell lines

A549, Colo320, HepG2, SW620, HT-29, PC-3, and K562 cells were kept in our laboratory (Hematological Laboratory of Zhujiang Hospital, Guangzhou, China) and genotyped to verify their authenticity. Cells were cultivated either in DMEM or RPMI 1640 (Invitrogen, Grand Island, NY, USA) with 10% fetal bovine serum (FBS; Invitrogen) and 1% penicillin/streptomycin.

### Molecular docking simulation in HLA-A24-restricted epitopes using AutoDock 4.2

We acquired crystal structures of the HLA-A24-peptide complexes available at the Protein Data Bank database (code: 2BCK). Then, we performed the molecular simulation study to predict the affinities between candidate peptides and the HLA-A24 protein via AutoDock software (version 4.2; The Scripps Research Institute, La Jolla, CA, USA). The grid center coordinate defined in the search space was determined according to that of the candidate peptide, and the size of the grid box was set to the default plus three points on each side of the cuboid. Prior to docking, all the water and solvent atoms of the protein were removed, and the Kollman charges were added. The Lamarckian Genetic Algorithm was employed as the docking algorithm with 50 runs in each of the 1×10^6^ energy evaluations. We obtained the mean of the top three affinities (i.e., the three lowest ΔG values) among the 50 runs as an affinity value of binding for each candidate peptide^36^.

### Induction of peptide-specific T cells

PBMCs from HLA-A*2402+ donors were separated with a Ficoll-Hypaque density gradient according to the standard protocols. Peptide-specific T cells were generated by repeated stimulation with the corresponding peptide. Briefly, the cells were incubated in RPMI 1640 with 10% FBS and 10 ng/mL IL-2 (Peprotech, Rock Hill, NJ, USA) at 37 °C with 5% CO_2_. On day 3, PBMCs were stimulated with peptides (10 μM). Half of the medium with 10 ng/mL IL-2 was changed every 2 days. After three cycles of in vitro stimulation, the peptide-specific T cells were established and subjected to functional tests^17,23^.

### In vitro cytotoxicity assays

Cytolytic activity was measured using a standard lactate dehydrogenase (LDH)-release assay (Sigma, St Louis, MO, USA) according to previously described protocols. Briefly, a constant number of target cells (5×10^3^ cells/well) were plated into 96-well plates. Peptide-sensitized CTLs were added at a ratio of 6.25:1, 12.5:1, 25:1, or 50:1 and incubated at 37 °C with 5% CO_2_ for 4 h. The percentage of specific lysis was determined according to the following formula: [(experimental LDH release−spontaneous LDH release)/(maximum LDH release−spontaneous LDH release)] × 100.

### Tetramer staining

HLA-peptide tetramers were produced by the Helixgen company (Guangzhou, China). Cells were stained with PE-tetramer, allophycocyanin (APC)-anti-CD3, and fluorescein isothiocyanate (FITC)-CD8 for 15 min. The cells were then resuspended in FACS buffer and analyzed by flow cytometry.

### IFN-γ production assays

A human IFN-γ ELISPOT kit (BD Pharmingen, San Jose, CA, USA) was used to determine the immunogenic potential of the peptides according to the manufacturer’s instructions. Briefly, K562 and K562/A24 cells were used as target cells and were pulsed with 10 μM corresponding peptide overnight suspended in medium supplemented with 1% FBS. Target cells were co-incubated with cultured PBMCs in a round-bottom 96-well plate for 20 h. Spots were captured and analyzed by an automated ELISPOT reader^37^.

### Measurement of cytokine secretion by T cells in response to cancer cells

ELISA was used to measure the levels of IL-2, TNF-α perforin, and granzyme B secreted by T cells using commercially available kits (Dakewe). HLA-A24+ CTLs were co-incubated with HT-29 cells at 1:1 E:T ratios in 200 μL of RPMI-1640 supplemented with 5% FBS in round-bottom 96-well plates for 24 h at 37 °C in 5% CO_2_ humidified air. The supernatants were collected after incubation, and the IL-2, TNF-α, granzyme B, and perforin production were examined according to the manufacturer’s instructions.

### Detection of CD69 and IFN-γ expression by flow cytometry

CTLs were stained with CD3, CD8 and CD69 anti-human mouse antibodies (mAbs), washed, and analyzed by flow cytometry (BD Biosciences, Franklin Lakes, NJ). The CD3+ CD8+ cells were gated and evaluated for activation cell subsets (CD69+). Briefly, T cells that were maintained under the same culture conditions but without any peptide stimulation were used as negative controls. CTLs were co-incubated at a 1:1 ratio with stimulator cells in round-bottom 96-well plates. After 1 h of incubation, 10 μg/mL Brefeldin A (eBioscience, San Diego, USA) was added to each well. After 5 h of additional incubation, the cells were centrifuged, washed, and stained with phycoerythrin (PE)-conjugated anti-CD3 (eBioscience) and peridinin-chlorophyll protein (PerCP)-conjugated anti-CD8 anti-human mAbs (Biolegend) for 30 min on ice in the dark. After surface staining, cells were washed, fixed, permeabilized, washed with Perm/Wash solution (BD Biosciences), stained with an anti-IFN-γ mAb (eBioscience), and analyzed by flow cytometry^38^.

### Cell proliferation/survival assays and apoptosis measurements

Adherent cells (2×10^3^/well) were plated in 96-well plates in complete medium in the presence or absence of peptides for 12 h. CCK-8 (Dojindo) assays were carried out according to the manufacturer’s instructions. Colony formation assays were also performed. Cells (500 cells/well) were seeded into six-well plates and treated with peptide 327, TAT-327, Scr-327, TAT-Scr or DMSO on the following day. After 2 weeks, the cells were fixed with 4% formaldehyde and washed with PBS when colonies were visible. Crystal violet was added to the plates to stain the colonies. The number of apoptotic cells was analyzed by flow cytometry (BD Bioscience) using Annexin V and propidium iodide (PI; BD Biosciences). Cells were exposed to 50 μM 327, TAT-327, Scr-327 or DMSO, and they were harvested and processed after 12 h according to the manufacturer’s instructions^39^.

### Wound healing assays and transwell assays

A549, Colo320, HepG2, SW620, HT-29, and PC-3/A24 cells were cultured in six-well plates at a density of 4×10^5^ cells/well. Wounds were made by 10-μL pipette tips in the confluent monolayers the following day. Cells were treated with DMSO, 327 or TAT-327 for 24 h. The wounds were photographed at 0, 12, and 24 h using an inverted microscope. For transwell assays, cells suspended in serum-free medium were seeded into the upper chambers of the transwell insert (24-well type, 8-µm pore size; Corning, 3422). The lower chambers were filled with medium containing 10% FBS. DMSO, 327, TAT-327 or TAT-Scr were added the following day. After 12 h of incubation, the migrated cells on the bottom chambers were fixed with 4% paraformaldehyde and stained with crystal violet. The number of migrated cells was counted under a bright field microscopy.

### FITC labeling of peptides and analysis of intracellular localization

Colo320 cells were pre-incubated in DMEM at 37 °C overnight for attachment before incubation with FITC-labeled peptides. After 2 h of incubation with the peptides, cells were rinsed twice with ice-cold PBS to remove the free and surface-bound FITC-labeled peptides, and the distribution of fluorescence was then observed by a confocal laser scanning microscope (ZEISS, LSM 880 with Airyscan), and images were captured.

### Western blotting analysis

In brief, after sample cells were prepared, total protein was extracted and subjected to sodium dodecyl sulphate-polyacrylamide gel electrophoresis. After transferring the separated proteins onto PVDF membranes (0.2 μm; Millipore, Billerica, MA, USA) and subsequent blocking, the membranes were immunoblotted with rabbit anti-human primary antibody overnight at 4 °C. After extensive washing, the membranes were incubated with horseradish peroxidase (HRP)-conjugated goat anti-rabbit IgG secondary antibody for 1 h at room temperature. This was followed by detection of the HRP signal with an enhanced chemiluminescence (ECL) substrate (Pierce biotechnology, Rockford, IL, USA).

### PBMC treatment in the mouse model

Female 4- to 6-week-old BALB/c nude mice were purchased and maintained under specific pathogen-free conditions. HT-29 cells (1×10^6^) from each group were injected subcutaneously into the flank region of mice to initiate tumorigenesis. After the tumor size reached approximately 50 mm^3^, mice were injected intratumorally with 1×10^7^ peptide-sensitized PBMCs in a final volume of 200 μL of PBS per injection once a week for 3 consecutive weeks. Tumors were monitored three times per week to observe tumor volumes in the different treatment groups of nude mice. Tumor samples were excised, fixed, and embedded in paraffin for immunohistochemical analyses^40,41^.

### Xenograft tumor model

HT-29 cells (1×10^6^) in 100 μL of PBS were subcutaneously inoculated to each BALB/c nude mouse. The treatments began after the size of the HT-29 tumors reached 50 mm^3^, and the treatments consisted of intraperitoneal (i.p.) injections of PBS, peptide 327, TAT-327, Scr-327 or TAT-Scr (50 mg/kg in 200 μL of PBS; *n* = 9 per group; we removed the maximal and minimal responder in each group at the end of the experiment). The treatment was performed every 3 days until eight treatments. Tumor growth was monitored by a digital caliper, and volume was calculated using the formula: (length × width^2^)/2^42^. Animals were killed after 24 days of treatment^32,43^.

### Statistical analysis

Statistical analyses were performed using Prism 5.0 software (GraphPad). Data were analyzed using unpaired Student’s *t* tests when comparing two experimental groups. *P* values less than 0.05 were considered significant.

## Electronic supplementary material

Supplementary Figure 1

Supplementary Figure 2

Supplementary Figure 3

Supplementary Table
