# Supplementary material for: A dual-function epidermal growth factor receptor pathway substrate 8 (Eps8)-derived peptide exhibits a potent cytotoxic T lymphocyte-activating effect and a specific inhibitory activity
Source: Cell Death Dis. 2018 Mar 7;9(3):379. doi: 10.1038/s41419-018-0420-5 (PMC5841361; doi:10.1038/s41419-018-0420-5)
Supplement: Supplementary file 1 — Supplementary Figure 1 [file 41419_2018_420_MOESM1_ESM.pdf]

Supplementary Figure 1

a

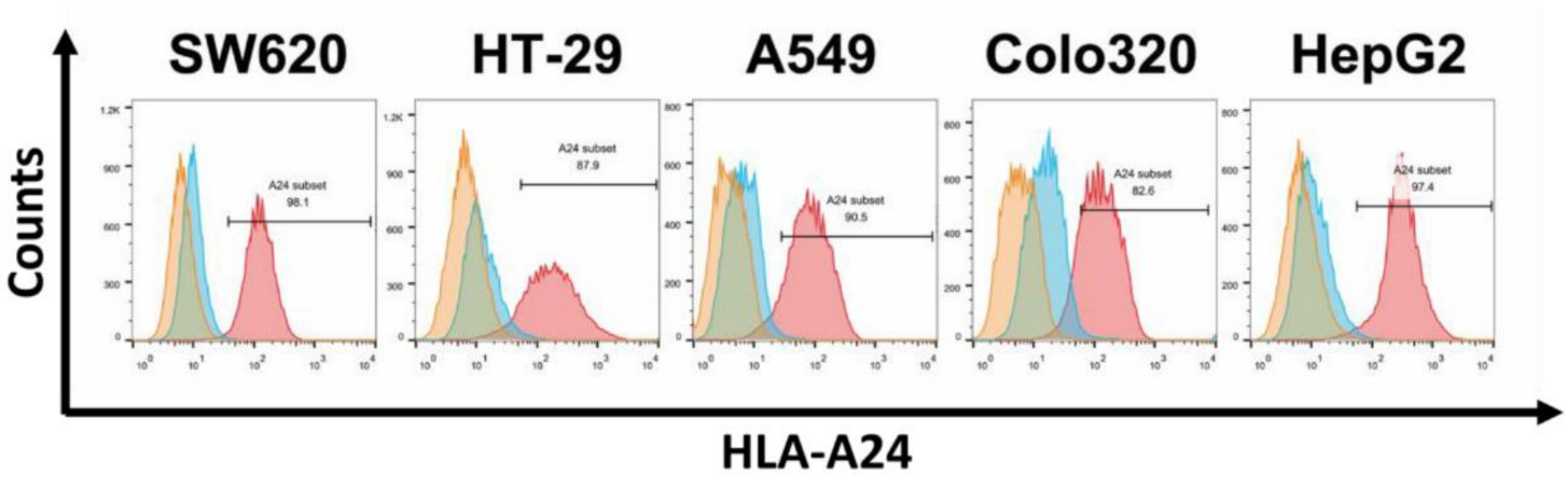

b

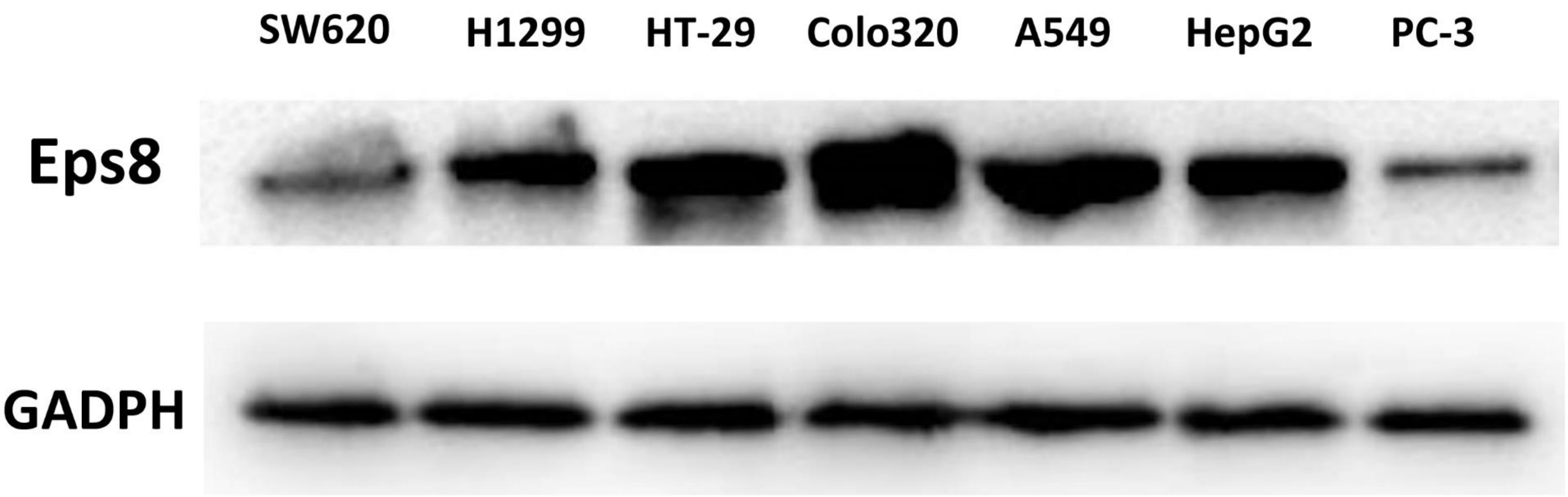

Supplementary figure 1 Characterization of the cancer cells we observed.

a, Analysis of HLA-A24 expression on different cancer cells. **b,** western blot detection of Eps8 protein levels in various cancer cell lines. The major bands at ~97 kDa, which corresponds to the calculated molecular weight for Eps8, are shown.
