# Supplementary material for: A dual-function epidermal growth factor receptor pathway substrate 8 (Eps8)-derived peptide exhibits a potent cytotoxic T lymphocyte-activating effect and a specific inhibitory activity
Source: Cell Death Dis. 2018 Mar 7;9(3):379. doi: 10.1038/s41419-018-0420-5 (PMC5841361; doi:10.1038/s41419-018-0420-5)
Supplement: Supplementary file 2 — Supplementary Figure 2 [file 41419_2018_420_MOESM2_ESM.pdf]

Supplementary Figure 2

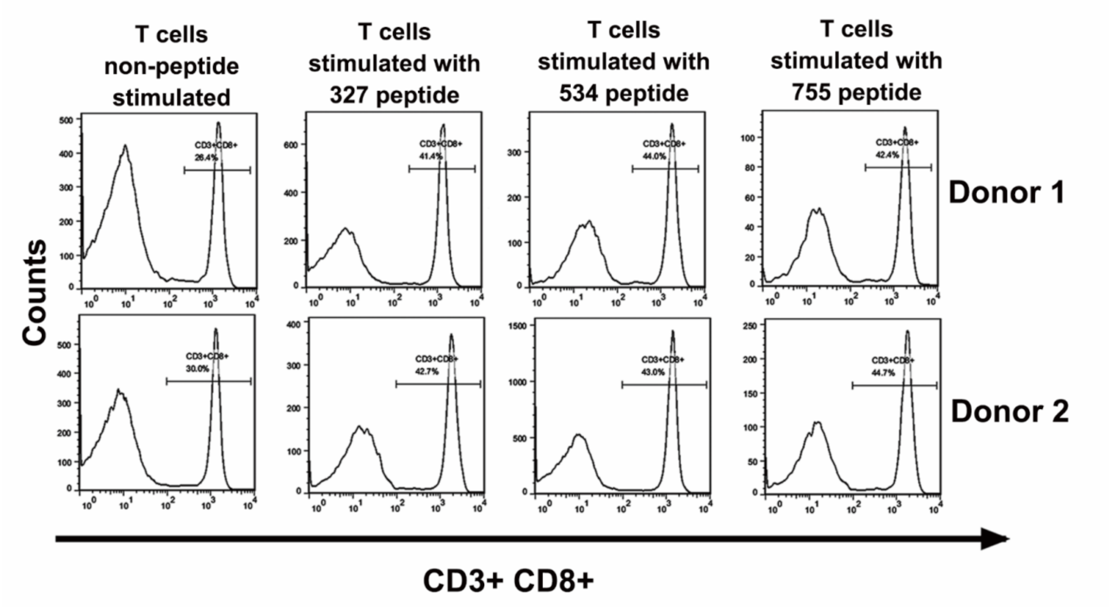

Supplementary Figure 2 The percentage of CD8+ T cells was increased in the CD3+ T cell cultures stimulated with the Eps8-specific peptide as compared to the control (non-peptide stimulated) cells
