# Supplementary material for: A dual-function epidermal growth factor receptor pathway substrate 8 (Eps8)-derived peptide exhibits a potent cytotoxic T lymphocyte-activating effect and a specific inhibitory activity
Source: Cell Death Dis. 2018 Mar 7;9(3):379. doi: 10.1038/s41419-018-0420-5 (PMC5841361; doi:10.1038/s41419-018-0420-5)
Supplement: Supplementary file 3 — Supplementary Figure 3 [file 41419_2018_420_MOESM3_ESM.pdf]

Supplementary Figure 3

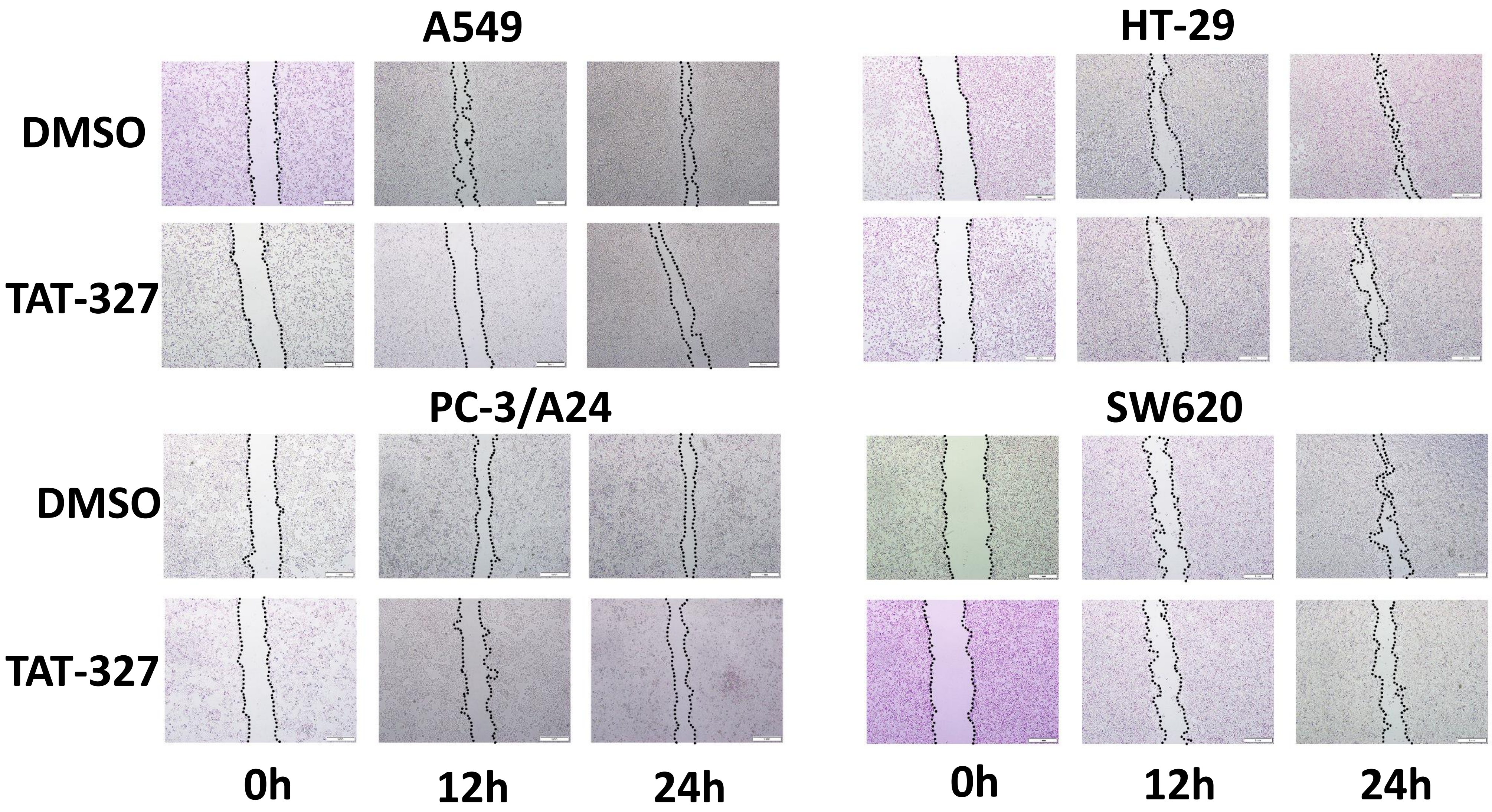

Supplementary Figure 3 The migration abilities were significantly reduced in peptide 327-treated cells in wound healing assay
