# Supplementary material for: A dual-function epidermal growth factor receptor pathway substrate 8 (Eps8)-derived peptide exhibits a potent cytotoxic T lymphocyte-activating effect and a specific inhibitory activity
Source: Cell Death Dis. 2018 Mar 7;9(3):379. doi: 10.1038/s41419-018-0420-5 (PMC5841361; doi:10.1038/s41419-018-0420-5)
Supplement: Supplementary file 4 — Supplementary Table [file 41419_2018_420_MOESM4_ESM.pdf]

Supplementary Table 1 HLA-A, and –DR genotypes of healthy donors analyzed in this study

**Donor ID**[a]	***HLA-A*** **genotype**	***HLA-DRB*** **genotype**
HD.1	*A*11:01 / 24:02*	*DRB1*09:01/11:01*
HD.2	*A*02:07 / 24:02*	*DRB1*08:03 / 09:01*
HD.3	*A*11:01 / 24:02*	*DRB1*15:01 / 15:01*
HD.4	*A*02:07 / 24:02*	*DRB1*03:01 / 08:03*
HD.5	*A*02:01 / 24:02*	*DRB1*07:01/ 09:01*

[a]PBMCs derived from healthy donors (HD.1 through HD.5) were used for the induction of Eps8-specific CTL cells.

Supplementary Table 2 HLA-A, and –DR genotypes of prostate cancer patients analyzed in this study

Donor ID[a]	*HLA-A* genotype	*HLA-DRB* genotype
PCC.1	*A*11:01 / 24:02*	*DRB1*04:05/12:02*
PCC.2	*A*24:02 / 33:03*	*DRB1*03:01 / 13:02*
PCC.3	*A*11:01 / 24:02*	*DRB1*11:01 / 15:01*
PCC.4	*A*11:01 / 24:02*	*DRB1*12:02 / 12:02*

[a]PBMCs derived from prostate cancer patients (PCC.1 through PCC.4) were used for the induction of Eps8-specific CTL cells.
